# Overlooked and Undernourished: A Case Report of Scurvy Linked to Food Insecurity

**DOI:** 10.5070/M5.52313

**Published:** 2026-04-30

**Authors:** Justin Kosley, Marshall Howell, Zachary Grant

**Affiliations:** * University of Texas Southwestern Medical Center, School of Medicine, Dallas, TX; ^ University of Texas Southwestern Medical Center, Department of Emergency Medicine, Dallas, TX

## Abstract

Food insecurity is a commonly underrecognized social determinant of health and is a risk factor for severe, though uncommon, nutritional deficiencies. This report describes a 60-year-old male with a history of asthma and housing instability who presented to the emergency department (ED) for shortness of breath and was found to have classic findings of vitamin C deficiency, colloquially known as scurvy. Examination revealed diffuse wheezing, poor dentition with palatal ecchymosis, and a perifollicular rash with scattered bruising on his bilateral lower extremities. Due to financial struggles, he was experiencing food and housing insecurity, with a diet primarily composed of processed carbohydrates. Laboratory studies revealed anemia, hypoalbuminemia, and severely low levels of vitamins C and B6. He was admitted for management of his asthma exacerbation and treatment of his nutritional deficiencies with vitamin supplementation, electrolyte repletion, and dietary support. Upon discharge, he was placed in a nearby shelter with financially accessible follow-up care, food bank resources, and multivitamin prescriptions. At follow-up three weeks later, his dermatologic findings had resolved.

This case highlights the importance of a thorough physical examination and detailed social history in the ED to avoid missing subtle yet clinically significant diagnoses. It underscores the need for early recognition and treatment of nutritional deficiencies, particularly in vulnerable populations. Furthermore, it demonstrates the value of accessible follow-up care in supporting the management of nutritional deficiencies and promoting long-term recovery.

**Topics:**

Scurvy, vitamin C deficiency, nutritional deficiency, nutritional insecurity, food insecurity.

**Figure:**
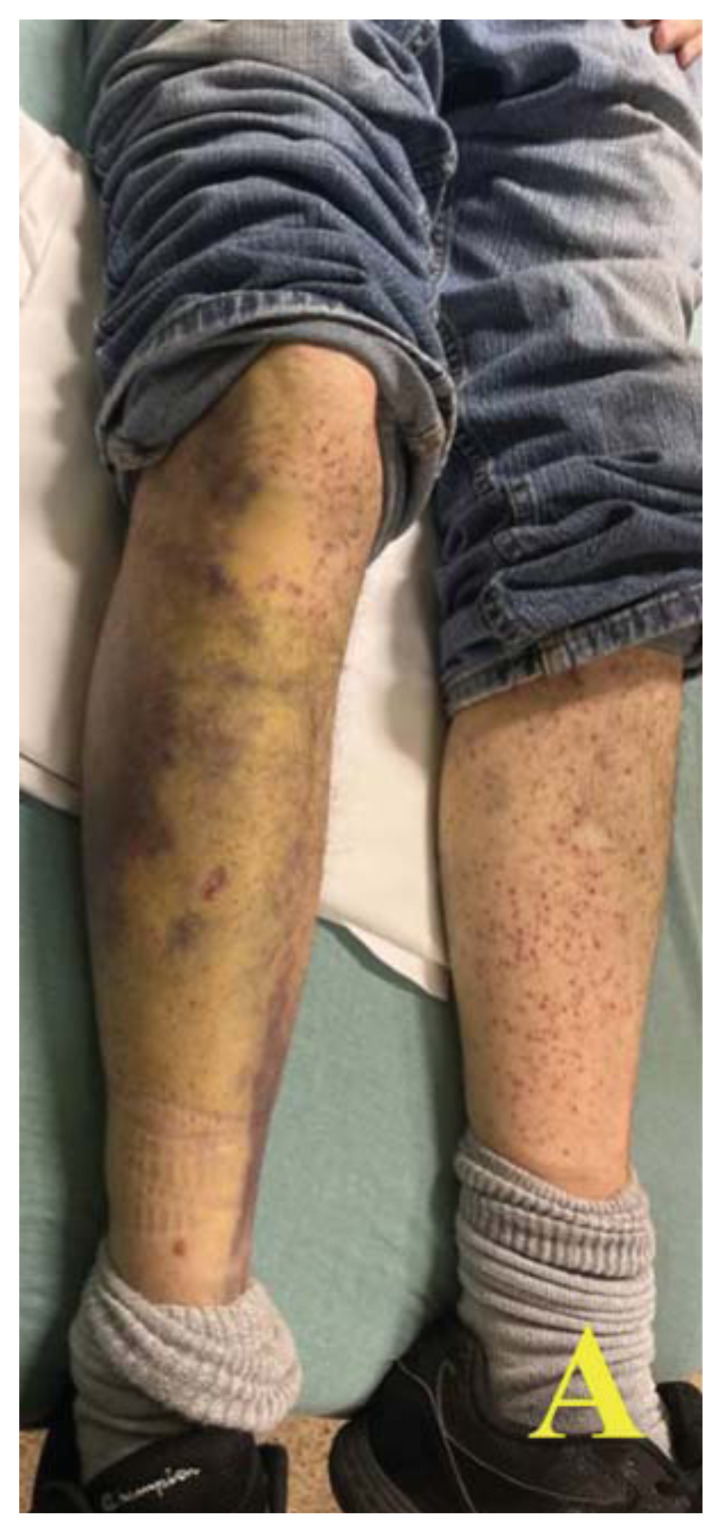


**Figure:**
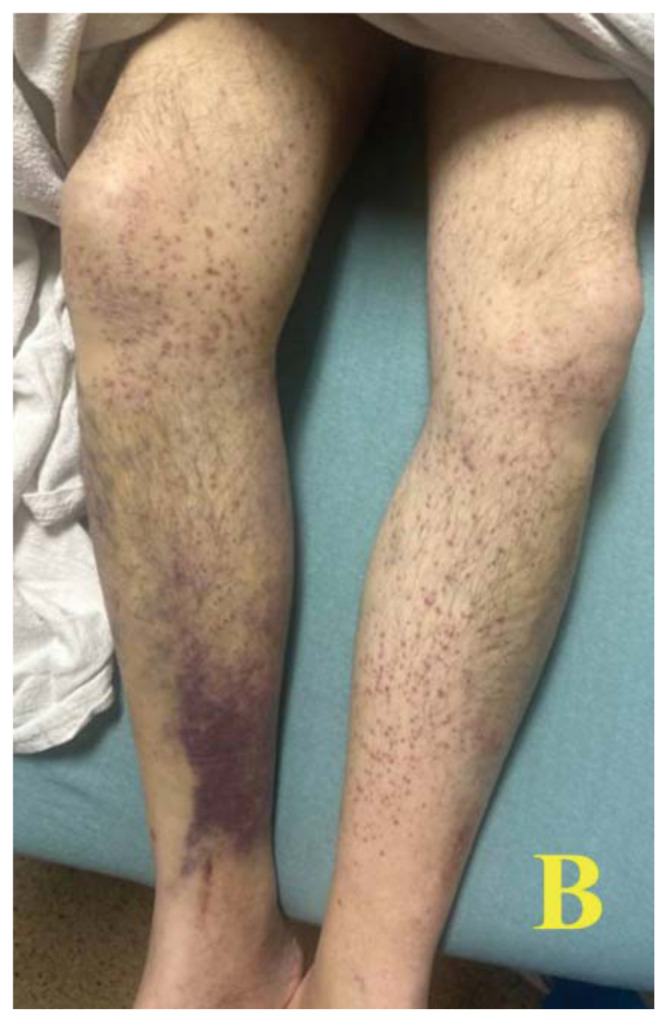


**Figure:**
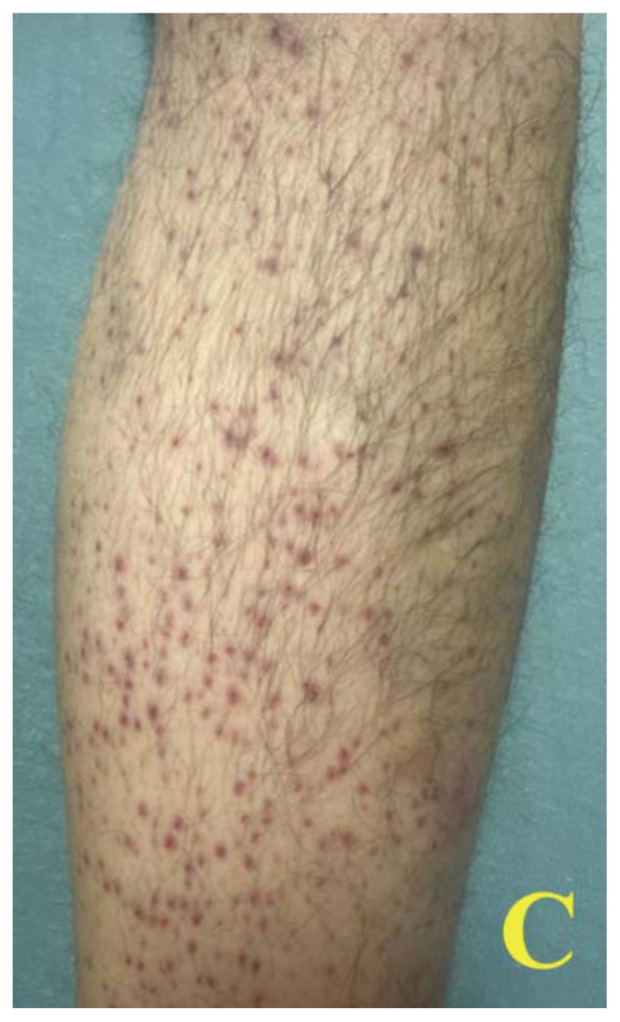


**Figure:**
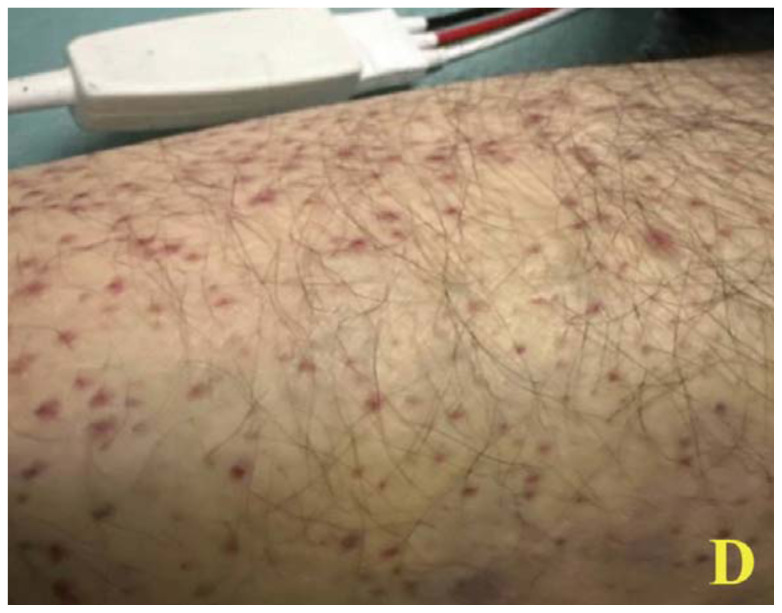


**Figure:**
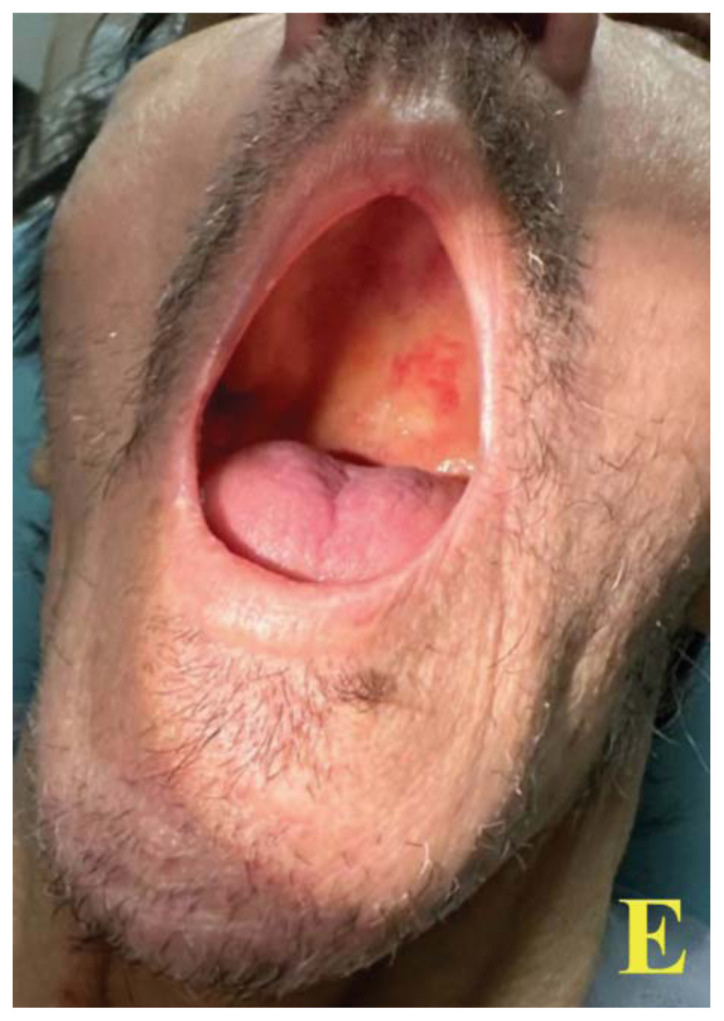


**Figure:**
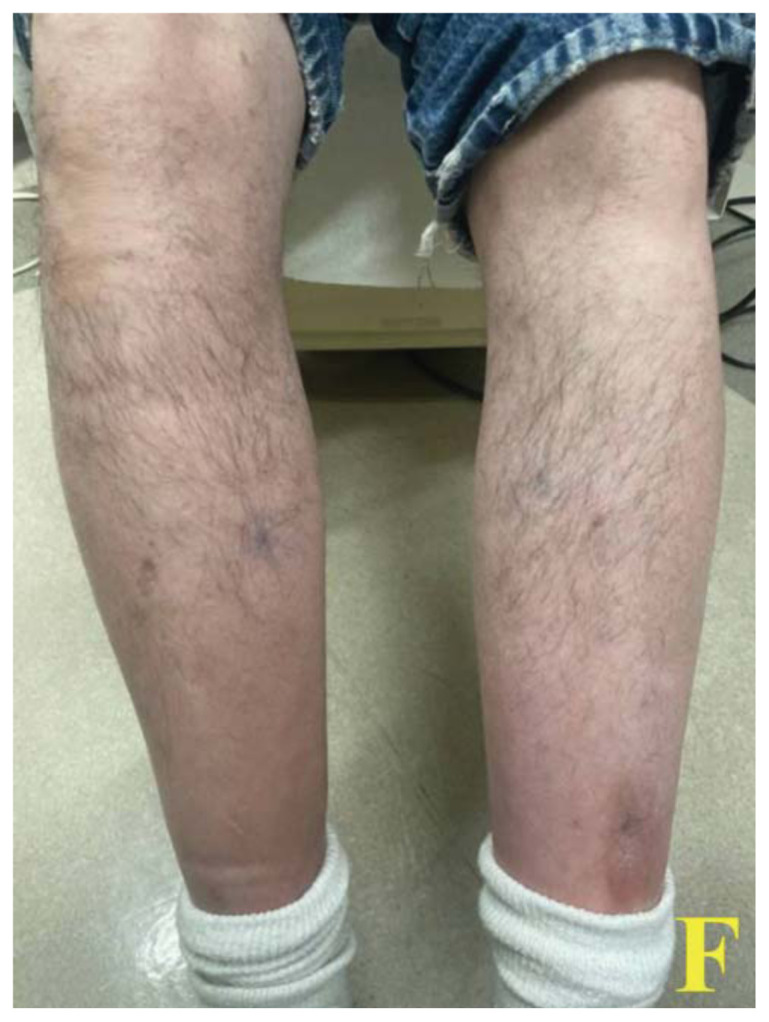


## Brief introduction

Ascorbic acid (vitamin C) plays a pivotal role in immune function and in maintaining the integrity of blood vessels and connective tissue by serving as a cofactor for prolyl and lysyl hydroxylases, which are required for stabilization of the triple-helical collagen structure.^1^ Humans are unable to synthesize ascorbic acid, making sufficient dietary intake from fruits, vegetables, or supplements essential. Deficiency impairs collagen fibril cross-linking, resulting in capillary fragility, defective wound healing, and musculoskeletal pain with manifestations developing within 1–3 months of inadequate intake.^2^ Vitamin C deficiency, also known as scurvy, may initially present with nonspecific symptoms such as arthralgias, myalgias, fatigue, and weight loss.^3^ As the disease progresses, pathognomonic findings can appear, including follicular hyperkeratosis with perifollicular hemorrhage, coiled or corkscrew hairs, gingival bleeding, and ecchymoses. If left untreated, scurvy can result in life-threatening systemic infection, hemolysis, and organ failure.^4^

Approximately 31% of the United States population is at risk for at least one vitamin deficiency or anemia.^5^ Research shows that the average American diet falls short of the estimated average requirements for vitamins A, C, D, and E, even with supplementation.^6^ These deficiencies often arise from poor dietary choices, food insecurity, or limited access to nutritious foods due to financial or social factors. In 2023, 18.0 million US households (13.5%) experienced food insecurity.^7^ Emergency physicians frequently encounter food insecure patients and must consider nutritional causes of illness, along with social determinants of health, when formulating differential diagnoses. In this report, we describe the case of an adult male who presented to the emergency department (ED) for shortness of breath and was incidentally found to have severe manifestations of vitamin C deficiency.

## Presenting concerns and clinical findings

A 60-year-old male with a past medical history of asthma presented to the ED via emergency medical services (EMS) for shortness of breath. On scene, EMS found the patient tachypneic with diffuse bilateral wheezes but without hypoxia. En route, they administered albuterol and ipratropium with supplemental oxygen, along with 10 mg intravenous (IV) dexamethasone. On arrival, the patient’s vital signs included a temperature of 36.5°C, heart rate of 76 beats/minute, blood pressure of 111/93 mm Hg, respiratory rate of 12 breaths/minute, and O^2^ saturation of 100% on room air. During the initial evaluation, the patient disclosed experiencing recent financial hardship that led to eviction from his apartment, inability to purchase essential medications, and severely restricted food intake for several weeks. On exam, he appeared pale and malnourished, with sunken eyes and poor dentition. He had ecchymosis on his hard palate and jaundice under his tongue, along with diffuse bilateral wheezes but no accessory muscle use. A skin exam revealed what appeared to be a painless petechial rash with scattered ecchymoses on his bilateral lower extremities. However, upon closer examination, these areas of petechiae were identified as perifollicular hemorrhages.

Lab work was remarkable for hemoglobin 7.7 g/dL (ref: 13.2–16.9 g/dL), potassium 2.9 mmol/L (ref: 3.6–5.0 mmol/L), albumin 3.2 g/dL (ref: 3.5–5.2 g/dL), total bilirubin 3.2 mg/dL (ref: 0.2–1.3 mg/dL), indirect bilirubin 2.5 mg/dL (ref: 0.2–0.8 mg/dL), vitamin B12 192 pg/mL (ref: ≥232 pg/mL). His mean corpuscular volume (MCV), platelet count, aspartate aminotransferase (AST), alanine aminotransferase (ALT), alkaline phosphatase, lactate dehydrogenase (LDH), haptoglobin, fibrinogen, magnesium, phosphorus, and calcium levels were all within normal range, and his Coombs test was negative. Vitamin C and B6 levels, along with iron studies, were also obtained but had not resulted during the patient’s ED stay. The patient was admitted for management of his acute asthma exacerbation and for further evaluation and treatment of presumed vitamin C deficiency.

## Significant findings

Images A and B of the lower extremities show what initially appeared to be a petechial rash with scattered ecchymoses bilaterally, primarily on the right lower leg. On closer examination, images C and D more clearly show these spots to be flat and non-blanching, indicative of follicular hyperkeratosis with perifollicular hemorrhages. The classic coiled or corkscrew hair findings are not seen in these images. Image E shows evidence of palatal ecchymosis, without evidence of gingival bleeding, likely due to loss of dentition. This constellation of exam findings reflects the production of weakened or dysfunctional collagen in connective tissue and vascular structures and is strongly suggestive of the clinical diagnosis of scurvy. Image F shows resolution of the patient’s dermatologic findings after three weeks of treatment.

## Patient course

On admission, the patient received standard treatment for his asthma exacerbation, including scheduled albuterol nebulizers and a course of steroids, resulting in resolution of his respiratory symptoms. He also underwent a workup for nutritional and vitamin deficiencies. Dermatology consultants evaluated the patient and confirmed the symptoms were related to vitamin C deficiency after consideration of a broad differential diagnosis. Rheumatologic causes seemed unlikely since the lesions were not raised or palpable. Without fever, erythema, induration, or warmth, infection was less likely. The clinical picture was also inconsistent with vasculitis without systemic findings such as renal, pulmonary, or gastrointestinal involvement. A normal platelet count and coagulation profile reduced concern for an underlying coagulopathy. Hemolysis was considered due to his indirect hyperbilirubinemia but was not supported by reassuring LDH, haptoglobin, fibrinogen, direct antiglobulin test (DAT), and iron studies. The indirect hyperbilirubinemia was likely attributable to impaired conjugation caused by the physiologic stress of malnutrition, severe dehydration, and multiple nutritional deficiencies. This diagnostic reasoning was supported when serum assays returned, revealing a vitamin C level of <0.1 mg/dL (reference: 0.4–2.0 mg/dL).

A registered dietitian was consulted to assist with repletion of the patient’s nutritional and vitamin deficiencies to minimize risk of refeeding syndrome. He was given IV vitamin C, folate, and thiamine, along with IM daily vitamin B12. Throughout his six-day hospitalization, his nutritional and vitamin deficiencies showed steady improvement both symptomatically and on laboratory evaluation.

At discharge, the patient was given prescriptions for oral vitamins C and D2 and vitamin B12 injections, and housing at a local shelter. He was provided placement in a shelter and follow-up with the hospital’s mobile community clinic for the unhoused. He received regular meals while staying at the shelter. At his clinic visit three weeks post-discharge, his dermatologic symptoms had resolved.

## Discussion

Scurvy is a clinical diagnosis in the emergency department. Due to the condition’s rarity, the sensitivity and specificity of the pictured exam findings are unknown, though their constellation is considered pathognomonic.^1^ The primary utility of laboratory testing in the workup of suspected scurvy is to rule out alternative diagnoses, including hematologic disorders, vasculitides, infections, and drug-related causes. Serum vitamin C levels may take several days to result, depending on local lab capabilities, making them less useful to the emergency physician. It is not uncommon for these patients to have additional vitamin and mineral deficiencies; therefore, obtaining levels of iron, thiamine, and vitamin B12 during hospitalization can be beneficial for their workup.

Treatment consists of supplementation and dietary modification, when feasible. For adults diagnosed with scurvy, 500 to 1000 mg/day of vitamin C is recommended for one month, with symptom improvement often observed within a few days.^2^ Oral supplementation is often sufficient, but in this case, the patient received IV vitamin C during hospitalization. This treatment is typically reserved for severe deficiencies due to its high bioavailability and rapid repletion rates, even in malabsorptive and refeeding states.^8^

In patients experiencing food and housing insecurity, reliable post-hospitalization follow-up care and monitoring can be challenging. A multidisciplinary approach to providing the patient with appropriate resources, education, and counseling is critical to ensuring long-term treatment success. In this case, the patient was discharged to a local shelter, where he could receive regular meals, housing, and follow-up medical care at minimal personal cost through the county healthcare system. Chart review indicated that his clinical manifestations of scurvy had resolved within three weeks of discharge with supplementation and improved nutrition.

Despite the availability of fortified foods and dietary supplements, nutritional deficiencies still exist in the United States today, primarily due to socioeconomic disparities. Food insecurity is associated with significantly higher rates of inadequate micronutrient intake in both men and women. Nearly half of food insecure adults (49% of men, 42% of women) have inadequate vitamin C intake, compared with 37% of men and 29% of women who are food secure. Similar disparities are observed for magnesium, zinc, vitamin B6, and vitamin D.^9^ This case highlights the importance of conducting a targeted and thoughtful history and physical examination for all patients. Emergency physicians must be attuned to the social needs of their patients and recognize how these factors influence their physical health. Thus, emergency physicians must maintain a broad differential diagnosis and conduct thorough, unbiased assessments to avoid missing subtle, yet clinically significant conditions.

## Supplementary Information
























