# Supplementary figures and images for: Overlooked and Undernourished: A Case Report of Scurvy Linked to Food Insecurity
Source: J Educ Teach Emerg Med. 2026 Apr 30;11(2):V48–52. doi: 10.5070/M5.52313 (PMC13152377; doi:10.5070/M5.52313)

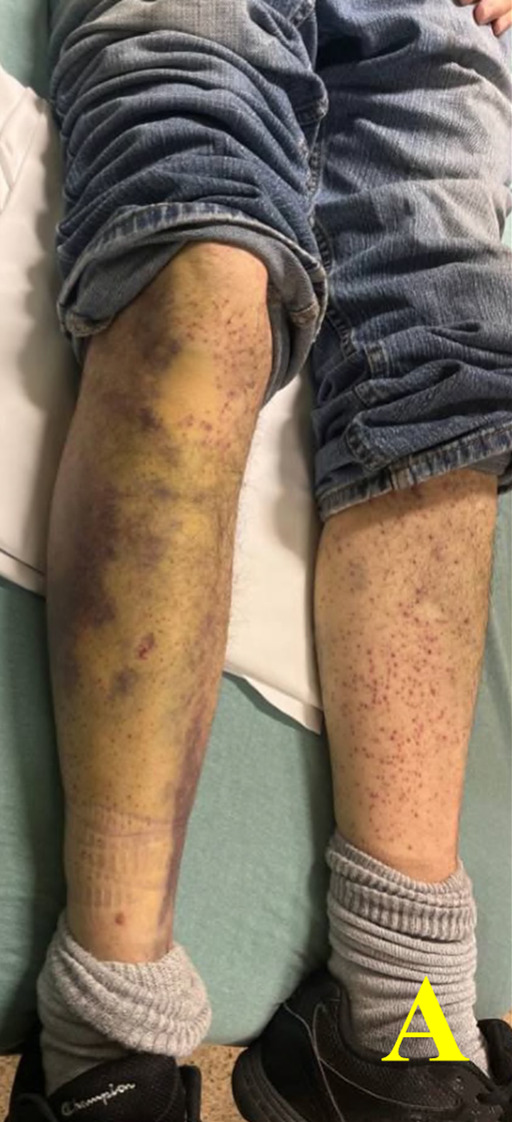

Supplement: Supplementary file 1 [file 11-2-V48-Supp1.jpg]

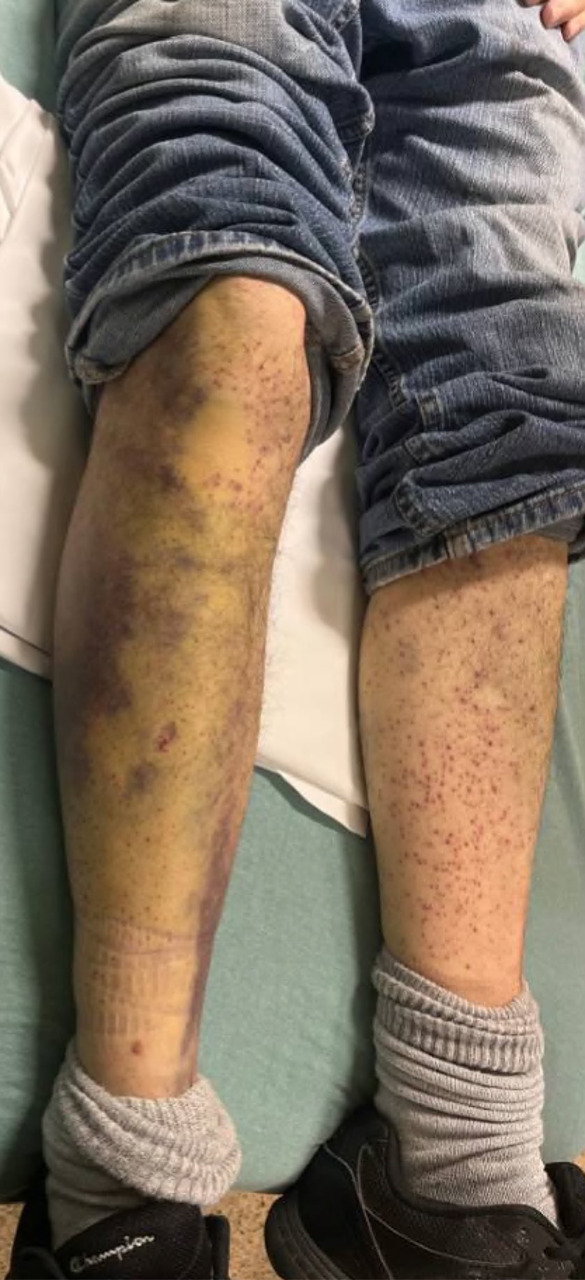

Supplement: Supplementary file 2 [file 11-2-V48-Supp2.jpg]

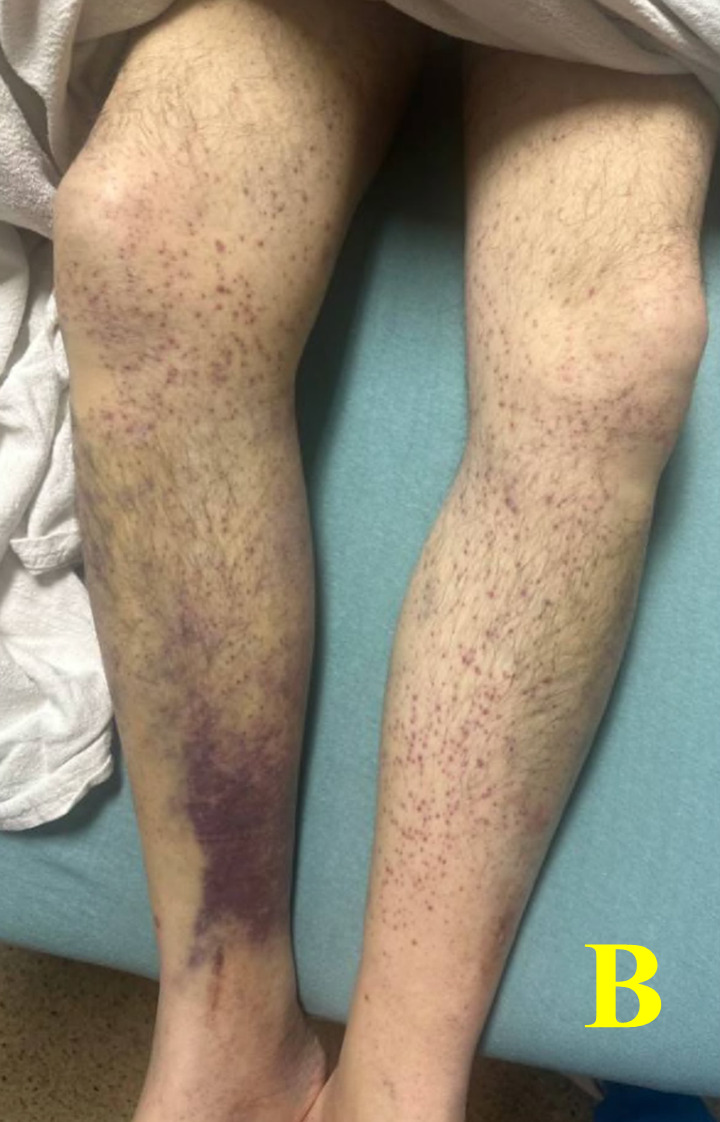

Supplement: Supplementary file 3 [file 11-2-V48-Supp3.jpg]

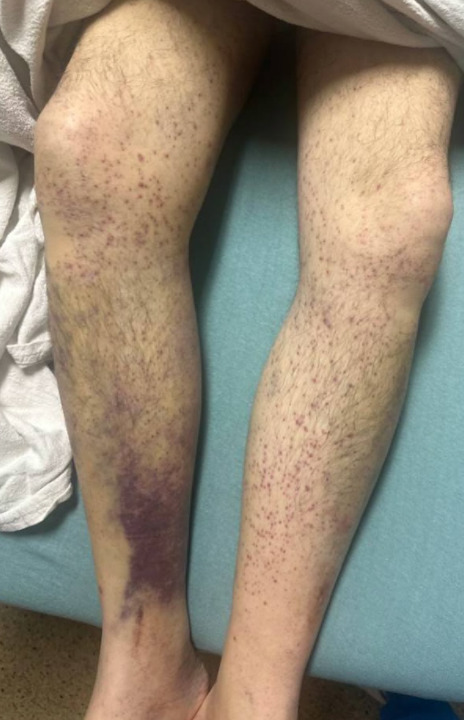

Supplement: Supplementary file 4 [file 11-2-V48-Supp4.jpg]

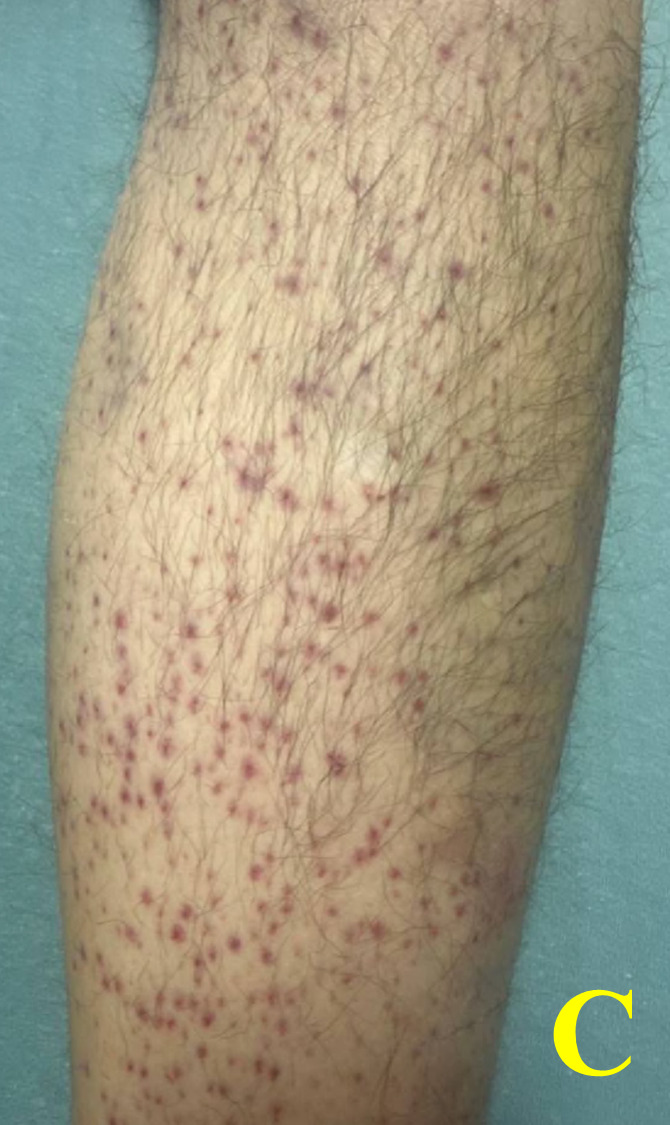

Supplement: Supplementary file 5 [file 11-2-V48-Supp5.jpg]

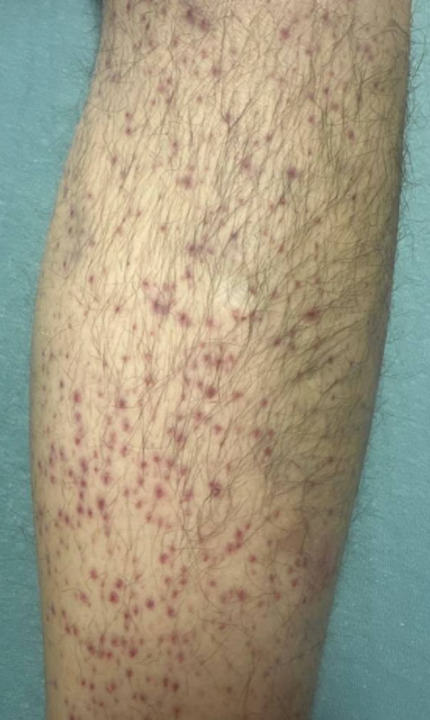

Supplement: Supplementary file 6 [file 11-2-V48-Supp6.jpg]

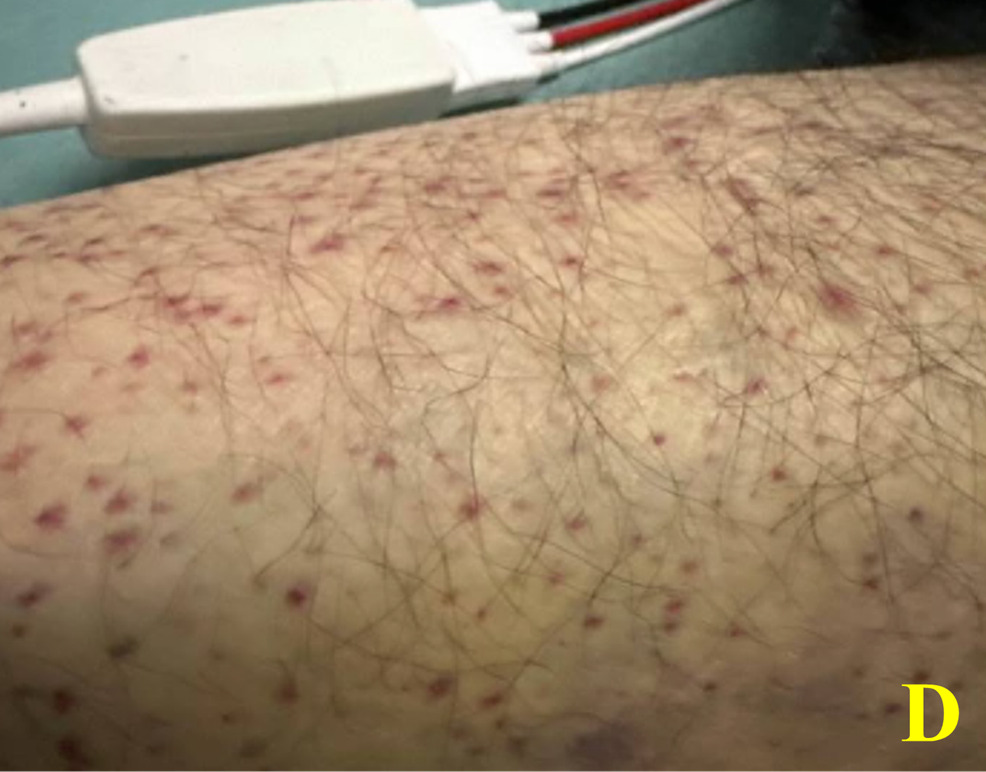

Supplement: Supplementary file 7 [file 11-2-V48-Supp7.jpg]

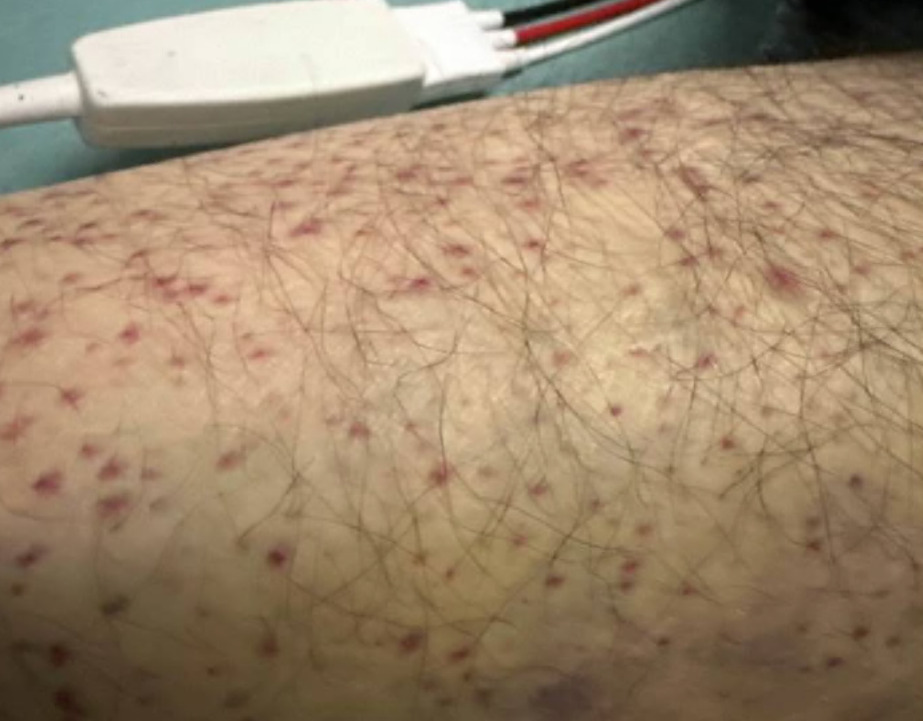

Supplement: Supplementary file 8 [file 11-2-V48-Supp8.jpg]

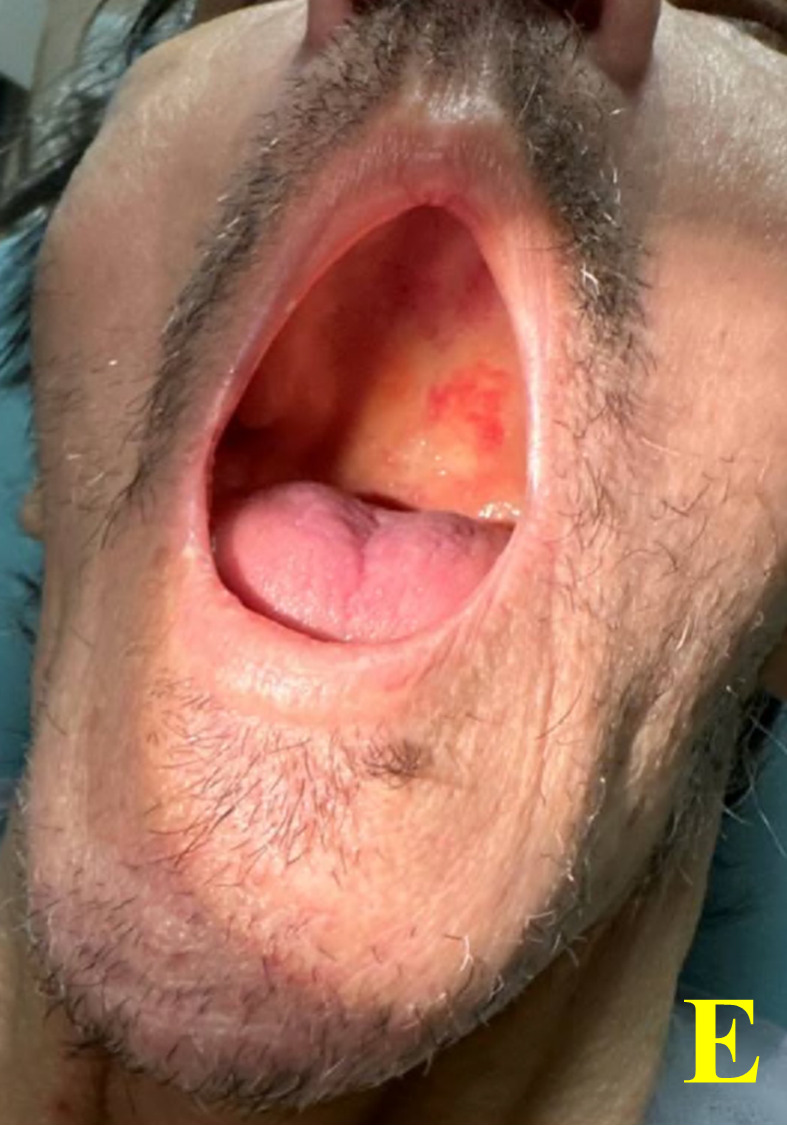

Supplement: Supplementary file 9 [file 11-2-V48-Supp9.jpg]

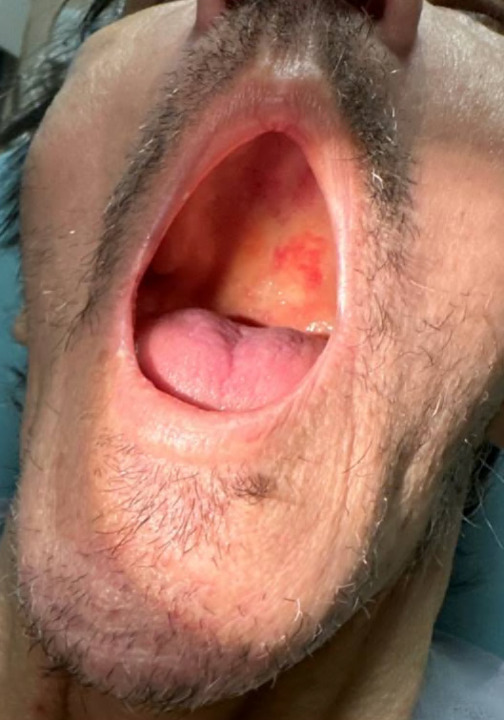

Supplement: Supplementary file 10 [file 11-2-V48-Supp10.jpg]

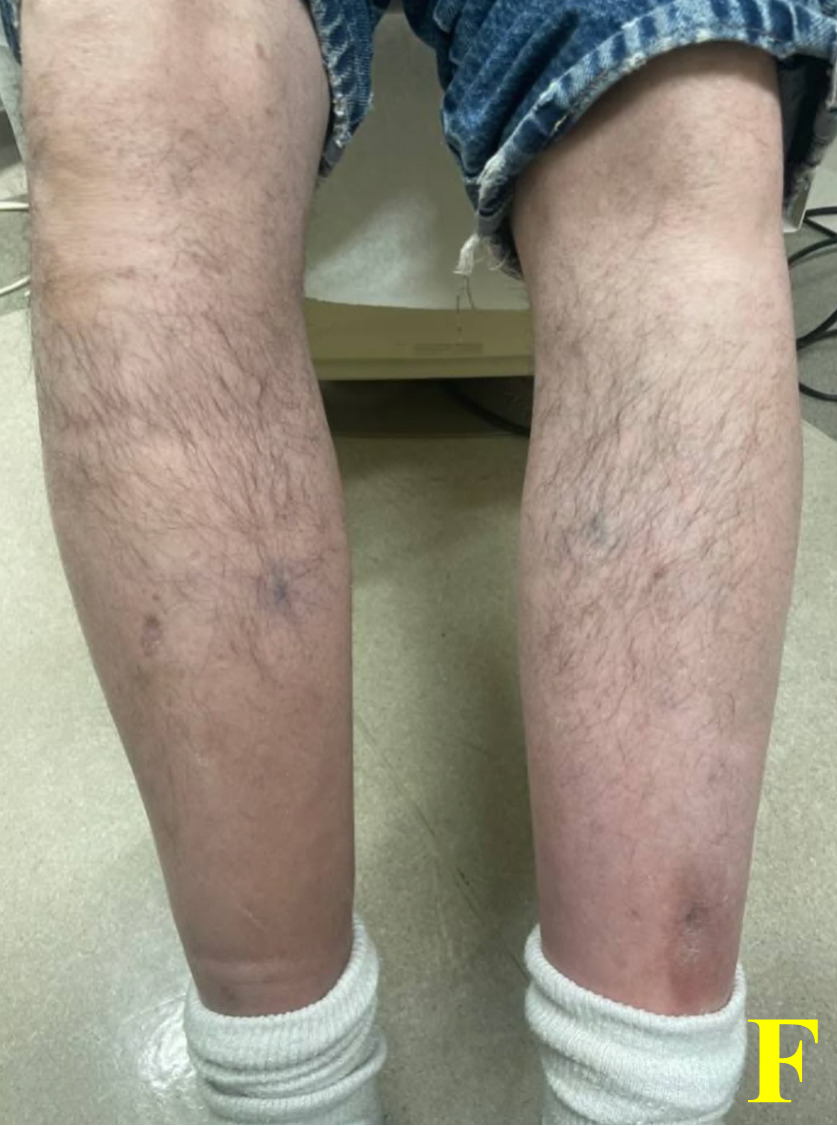

Supplement: Supplementary file 11 [file 11-2-V48-Supp11.jpg]

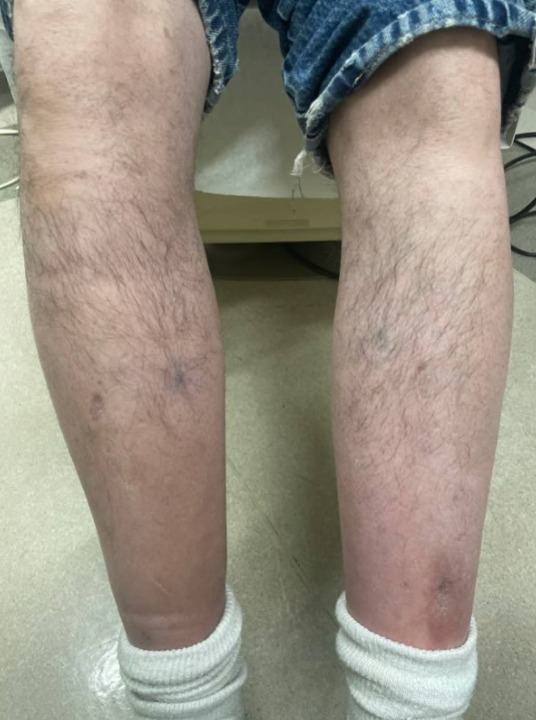

Supplement: Supplementary file 12 [file 11-2-V48-Supp12.jpg]
